# Avanços em Estresse Oxidativo nas Doenças Cardiovasculares

**DOI:** 10.36660/abc.20250478

**Published:** 2025-09-04

**Authors:** Gabriela Brandão, Carolina R. Tonon, Marina P. Okoshi

**Affiliations:** 1 Universidade Estadual Paulista Faculdade de Medicina de Botucatu Departamento de Clínica Médica Botucatu SP Brasil Departamento de Clínica Médica – Faculdade de Medicina de Botucatu – Universidade Estadual Paulista (UNESP), Botucatu, SP – Brasil

**Keywords:** Espécies Reativas de Oxigênio, Doenças Cardiovasculares, Fisiologia, Antioxidantes

As doenças cardiovasculares constituem as principais causas de morbimortalidade no mundo. O estresse oxidativo desempenha papel importante na patogênese e progressão de doenças cardiovasculares como a aterosclerose, isquemia miocárdica, insuficiência cardíaca e hipertensão arterial sistêmica e pulmonar.^1 , 2^

O estresse oxidativo é caracterizado pelo desequilíbrio entre a produção de radicais livres e as defesas antioxidantes. Radicais livres são moléculas com um elétron desemparelhado, que as torna altamente reativas e capazes de causar danos celulares. Os radicais livres mais comuns são as espécies reativas de oxigênio (ROS) e as espécies reativas de nitrogênio (RNS). As ROS incluem o ânion superóxido, o radical hidroxila e o peróxido de hidrogênio, enquanto as RNS incluem o óxido nítrico, o peroxinitrito e o dióxido de nitrogênio. Os radicais livres podem ser produzidos em diversos compartimentos celulares, como o citoplasma, membrana celular, retículo endoplasmático, mitocôndrias e peroxissomos, principalmente como resultado de reações enzimáticas.^3^

Em condições fisiológicas, as ROS são essenciais em várias funções celulares, incluindo sinalização intracelular e defesa contra patógenos. Por exemplo, a nicotinamida adenina dinucleotídeo fosfato reduzido (NADPH) oxidase, presente nas membranas de células fagocíticas, desempenha papel crítico na resposta imune. Quando ativada, a enzima transfere elétrons do NADPH para gerar o ânion superóxido e promover a destruição de patógenos.^4^ No entanto, excesso de ROS e RNS leva à modificação estrutural e funcional de proteínas, interrupção de vias de sinalização, danos oxidativos e nitrativos ao DNA (causando mutações e quebras de fita), peroxidação lipídica comprometendo a integridade da membrana e dano celular. Dessa forma, o estresse oxidativo pode induzir inflamação, apoptose, autofagia desregulada e disfunção mitocondrial.^5^

Estresse oxidativo e inflamação são processos estreitamente relacionados, ambos implicados no desenvolvimento e progressão das doenças cardiovasculares. As ROS e RNS estimulam o fator nuclear kappa B (NF-κB), fator de transcrição que regula positivamente marcadores inflamatórios como o fator de necrose tumoral alfa (TNF-α), interleucina-1 beta (IL-1β), interleucina-6 (IL-6) e interferon-gama (IFN-γ). Essas citocinas promovem apoptose de miócitos e células endoteliais, desregulação do trânsito intracelular de cálcio, ativação de fibroblastos cardíacos em miofibroblastos, redução da produção de óxido nítrico, recrutamento de monócitos para a parede vascular, acúmulo de LDL em macrófagos, migração de células musculares lisas para a íntima arterial e aumento da expressão de moléculas de adesão em células endoteliais ativadas. Todos esses fatores contribuem para fibrose, vasoconstrição e formação de placas, mecanismos básicos das doenças cardiovasculares.^6^

Enzimas antioxidantes como a glutationa peroxidase, superóxido dismutase e catalase atuam em conjunto com antioxidantes não enzimáticos como ácido úrico, melatonina, glutationa e poliaminas para neutralizar as ROS e RNS.^7^ A suplementação de antioxidantes exógenos, como polifenóis, flavonoides e carotenoides, substâncias abundantemente presentes em plantas, apresentaram potentes efeitos antioxidantes.^4^ Recentemente, efeitos antioxidantes do chá branco e de uma combinação de chá preto e verde, ambos ricos em compostos bioativos como flavonoides e xantinas, foram avaliados em camundongos tratados com angiotensina II. A suplementação com esses extratos atenuou a disfunção endotelial da aorta, a hipertrofia cardíaca, e o estresse oxidativo e apoptose induzidos por isquemia e reperfusão no tecido miocárdico.^8^ Modulação do estresse oxidativo também é um dos mecanismos de ação de diversos fármacos utilizados em doenças cardiovasculares. Por exemplo, os efeitos benéficos dos inibidores do cotransportador de sódio-glicose tipo 2 (iSGLT2) — em pacientes diabéticos e não diabéticos — são atribuídos, pelo menos em parte, à sua capacidade em reduzir o estresse oxidativo cardíaco e sistêmico.^9 , 10^

Em decorrência da forte associação entre estresse oxidativo e doenças cardiovasculares, realizamos revisão de artigos publicados recentemente sobre o tema nos *Arquivos Brasileiros de Cardiologia* . Neste editorial, comentamos cinco artigos publicados que abordam o estresse oxidativo em modelos clínicos e experimentais de doenças cardiovasculares.

Pederiva et al.^11^ mostraram que o hormônio tireoidiano triiodotironina (T3) exerce efeito vasorrelaxante e atividade antioxidante em anéis aórticos isolados de ratos e previamente contraídos por ação da fenilefrina. O hormônio reduziu a atividade da NADPH oxidase e aumentou a atividade da superóxido dismutase, indicando efeitos benéficos no equilíbrio redox vascular e no tônus vasomotor.^11^

Efeitos do hormônio tireoidiano no estado oxidativo também foram avaliados em ratos com hipertensão arterial pulmonar induzida por monocrotalina.^12^ A monocrotalina causa remodelação adversa de vasos pulmonares, aumentando a pós-carga do ventrículo direito, induzindo hipertrofia e insuficiência cardíaca. Suplementação com hormônio tireoidiano e suco de uva, isoladamente ou em combinação, modulou o estresse oxidativo por aumento da atividade da xantina oxidase, normalizou proteínas envolvidas no trânsito intracelular de cálcio, e reduziu a remodelação cardíaca.

Infarto do miocárdio em ratos é um modelo amplamente utilizado para investigar remodelação e insuficiência cardíaca.^7^ No entanto, os pulmões raramente são analisados nesses animais. Tasca et al.^13^ investigaram os efeitos do pterostilbeno nos pulmões de ratos infartados. O pterostilbeno é um composto antioxidante encontrado em uvas e mirtilos. Sua suplementação aumentou a atividade da superóxido dismutase e catalase e reduziu a peroxidação lipídica, um marcador de estresse oxidativo. O composto também aumentou a expressão do fator nuclear relacionado ao eritroide 2 (Nrf2), proteína que atua como fator de transcrição com papel importante na síntese de enzimas antioxidantes endógenas. Em condições fisiológicas, o Nrf2 está ancorado à proteína associada ao Keap1. Quando há alteração da homeostase redox, o complexo Nrf2-Keap1 se dissocia, liberando o Nrf2, que se desloca para o núcleo e induz a transcrição de moléculas antioxidantes. Estudos adicionais são necessários para verificar o potencial de translação desses resultados para outras condições experimentais e clínicas.

O fator de diferenciação de crescimento 15 (GDF-15) é uma citocina associada ao estresse oxidativo e à inflamação. Sua concentração foi negativamente correlacionada à disfunção renal em pacientes com insuficiência cardíaca.^14^ Esse achado permite levantar a hipótese que o GDF-15 pode ser não apenas um fator determinante do prognóstico, mas também modulador da fisiopatologia da insuficiência cardíaca.

Finalmente, revisão sistemática avaliou os benefícios de quatro semanas de treinamento resistido no coração de roedores alimentados com dieta hipercalórica, em comparação a controles não treinados. A revisão incluiu estudos originais de dezembro de 2007 a dezembro de 2022. Os resultados mostraram que o treinamento atenua o estresse oxidativo, a inflamação e o estresse do retículo endoplasmático. No entanto, atenuação do estresse oxidativo não foi acompanhada por alterações histomorfométricas ou funcionais dos cardiomiócitos.^15^

Em conclusão, a investigação de vias indutoras de estresse oxidativo como marcadores prognósticos ou alvos terapêuticos no tratamento de doenças cardiovasculares parece ser promissora. No entanto, mais estudos são necessários para viabilizar a translação desses achados para a prática clínica.
