# Supplementary material for: Avanços em Estresse Oxidativo nas Doenças Cardiovasculares
Source: Arq Bras Cardiol. 2025 Sep 4;122(8):e20250478. [Article in Portuguese] doi: 10.36660/abc.20250478 (PMC12677806; doi:10.36660/abc.20250478)
Supplement: Translation

# Translation: Recent Advances in Oxidative Stress and Cardiovascular Diseases

**DOI:** 10.36660/abc.20250478i

**Authors:** Gabriela Brandão, Carolina R. Tonon, Marina P. Okoshi

**Affiliations:** 1 Botucatu SP Brazil Departamento de Clínica Médica – Faculdade de Medicina de Botucatu – Universidade Estadual Paulista (UNESP), Botucatu, SP – Brazil

**Keywords:** Reactive Oxygen Species, Cardiovascular Diseases, Physiology, Antioxidants

Cardiovascular diseases are the leading causes of morbidity and mortality worldwide. Oxidative stress plays an important role in the pathogenesis and progression of cardiovascular diseases such as atherosclerosis, myocardial ischemia, heart failure, and systemic and pulmonary arterial hypertension.^1 , 2^

Oxidative stress is characterized by an imbalance between free radical production and antioxidant defenses. Free radicals are molecules with an unpaired electron, making them highly reactive and capable of causing cellular damage. The most common free radicals are reactive oxygen species (ROS) and reactive nitrogen species (RNS). ROS include superoxide, hydroxyl radical, and hydrogen peroxide, whereas RNS comprise nitric oxide, peroxynitrite, and nitrogen dioxide. Free radicals can be produced in various cellular compartments, such as the cytoplasm, cell membrane, endoplasmic reticulum, mitochondria, and peroxisomes, mainly resulting from enzymatic reactions.^3^

Under physiological conditions, ROS are essential in several cell functions, including intracellular signaling and pathogen defense. For example, the nicotinamide adenine dinucleotide phosphate reduced (NADPH) oxidase, present in the membranes of phagocytic cells, plays a critical role in immune response. Once activated, it transfers electrons from NADPH, generating superoxide and enhancing pathogen destruction.^4^However, ROS and NOS in excess lead to structural and functional protein modification, disruption of signaling pathways, oxidative and nitrative DNA damage, causing mutations and strand breaks, lipid peroxidation, compromising membrane integrity, and cell damage. Therefore, oxidative stress can induce inflammation, apoptosis, dysregulated autophagy, and mitochondrial dysfunction.^5^

Oxidative stress and inflammation are two closely interconnected processes, both implicated in the development and progression of cardiovascular diseases. ROS and RNS stimulate the nuclear factor kappa B (NF-κB), a transcriptional factor that upregulates inflammatory markers such as tumor necrosis factor-alpha (TNF-α), interleukin-1 beta (IL-1β), interleukin-6 (IL-6), and interferon-gamma (IFN-γ). These cytokines promote apoptosis of myocytes and endothelial cells, dysregulation of calcium transient, activation of cardiac fibroblasts into myofibroblasts, reduction in nitrous oxide production, recruitment of monocytes into the vessel wall, accumulation of low-density lipoprotein (LDL) in macrophages, migration of smooth muscle cells to the arterial intima, and increased expression of adhesion molecules on activated endothelial cells. All these factors contribute to fibrosis, vasoconstriction, and plaque formation, which are basic mechanisms of cardiovascular diseases.^6^

Antioxidant enzymes such as glutathione peroxidase, superoxide dismutase, and catalase work in concert with non-enzymatic antioxidants like uric acid, melatonin, glutathione, and polyamines to neutralize ROS and RNS.^7^Among exogenous antioxidants, polyphenols, flavonoids, and carotenoids—abundantly found in natural plant sources—have shown potent antioxidant effects when supplemented.^4^Recently, the antioxidant effects of white tea and a combination of black and green tea—both rich in bioactive compounds such as flavan-3-ols and xanthines—were evaluated in angiotensin II-treated mice. Supplementation with both tea extracts attenuated aortic endothelial dysfunction, cardiac hypertrophy, and ischemia–reperfusion-induced oxidative stress and apoptosis in myocardial tissue.^8^Modulation of oxidative stress is also a mechanism of different drugs used in cardiovascular diseases. For example, the beneficial effects of sodium-glucose cotransporter 2 inhibitors (SGLT2i)—in both diabetic and non-diabetic patients—are at least partly attributed to their ability to reduce cardiac and systemic oxidative stress.^9 , 10^

Given the link between oxidative stress and cardiovascular diseases, we conducted a review of recently published articles on this topic in the *Arquivos Brasileiros de Cardiologia* . In this editorial, we comment on five articles that are related to oxidative stress and cardiovascular diseases in both clinical and experimental models.

Pederiva et al.^11^showed that triiodothyronine (T3) exerts a vasorelaxant effect and antioxidant activity in rat isolated aortic rings precontracted with phenylephrine. T3 reduced NADPH oxidase activity and increased superoxide dismutase activity, indicating beneficial effects on vascular redox balance and vasomotor tone.^11^

The effect of thyroid hormone on oxidative status was also evaluated in rats with monocrotaline-induced pulmonary arterial hypertension.^12^Monocrotaline causes adverse remodeling of lung vessels, increasing right ventricular afterload, and inducing right ventricular hypertrophy and heart failure. Supplementation of thyroid hormone and grape juice, either alone or in combination, modulated oxidative stress by increasing xanthine oxidase activity, normalized calcium-handling proteins, and reduced cardiac remodeling.

Myocardial infarction in rats is a widely used model to investigate cardiac remodeling and heart failure.^7^However, lungs have not been often analyzed in infarcted rats. Tasca et al.^13^investigated the effects of pterostilbene on the lungs of infarcted rats. Pterostilbene is an antioxidant compound found in grapes and blueberries. Its supplementation increased superoxide dismutase and catalase activities and reduced lipid peroxidation levels, a marker of oxidative stress. It also upregulated the expression of the nuclear factor erythroid 2-related factor 2 (Nrf2), a protein that acts as a transcription factor, with an important role in the synthesis of endogenous antioxidant enzymes. Under physiological redox status, Nrf2 is anchored to Kelch-like ECH-associated protein 1 (Keap1). When redox homeostasis is disrupted, the Nrf2-Keap1 complex dissociates and releases Nrf2, which translocates to the nucleus and induces the transcription of antioxidant molecules. Additional studies are needed to verify the potential for translating these results to other conditions.

The growth differentiation factor-15 (GDF-15) is a cytokine associated with oxidative stress and inflammation. Their levels were negatively correlated with renal dysfunction in heart failure patients.^14^This fact allows us to hypothesize that GDF-15 may not only be a prognostic factor, but also a modulator of heart failure pathophysiology.

Finally, a systematic review evaluated the benefits of four weeks of resistance training on the hearts of rodents fed a high-calorie diet compared with non-exercised controls. The review included original studies from December 2007 to December 2022. Results showed that training attenuated oxidative stress, inflammation, and endoplasmic reticulum stress. However, attenuation in oxidative stress was not accompanied by changes in histomorphometric features or cardiomyocyte contractile function.^15^

In conclusion, exploring oxidative stress pathways both as markers for cardiac prognosis and as therapeutic targets for treating cardiovascular diseases appears promising. Nevertheless, more studies are needed to support their translation into clinical practice.
